# Influence of measurement principle on total hemoglobin value

**DOI:** 10.1186/s12871-020-00991-2

**Published:** 2020-04-07

**Authors:** Keisuke Hayashi, Takashi Hitosugi, Yoshifumi Kawakubo, Norihisa Kitamoto, Takeshi Yokoyama

**Affiliations:** 1 Department of Dental Anesthesiology, Faculty of Dental Science, Kyushu University, 3-1-1 Maidashi, Higashi-ku, Fukuoka, 812-8582 Japan; 2 Clinical Engineering, Okayama saiseikai General Hospital, 2-25, Kokutai-cho, Kita-ku, Okayama, 700-8511 Japan

**Keywords:** Total hemoglobin (tHb), Blood gas analysis device, Absorbance measurement, Conductivity measurement

## Abstract

**Background:**

Total hemoglobin (tHb) measurement is indispensable for determining the patient’s condition (hemorrhagic vs. ischemic) and need for blood transfusion. Conductivity- and absorbance-based measurement methods are used for blood gas analysis of tHb. For conductivity-based measurement, tHb is calculated after converting blood conductivity into a hematocrit value, whereas absorbance measurement is based on light absorbance after red blood cell hemolysis. Due to changes in plasma electrolytes and hemolysis, there is a possibility that conductivity- and absorbance-based measurement methods may cause a difference in tHb.

**Methods:**

In this study, test samples with controlled electrolyte changes and hemolysis were created by adding sodium chloride, distilled water or hemolytic blood to blood samples collected from healthy volunteers, and tHb values were compared between both methods.

**Results:**

Conductivity-based measurement revealed reduced tHb value (from 15.49 to 13.05 g/dl) following the addition of 10% sodium chloride, which was also reduced by the addition of hemolysate. Conversely, the addition of distilled water significantly increased tHb value than the expected value. In the absorbance method, there was no significant change in tHb value due to electrolyte change or hemolysis.

**Conclusions:**

We have to recognize unexpected conductivity changes occur at all times when tHb is measured via conductivity- and absorbance-based measurement methods. The absorbance method should be used when measuring tHb in patients with expected blood conductivity changes. However, when using this method, the added contribution of hemoglobin from hemolytic erythrocytes lacking oxygen carrying capacity must be considered. We recognize that discrepancy can occur between conductivity- and absorbance-based measurement methods when tHb is measured.

## Background

The total hemoglobin (tHb) value in red blood cells (RBCs) is a critical measure of blood oxygen transport capacity. When the hemoglobin value abruptly declines due to hemorrhage, oxygenation of the tissue cannot be properly maintained. Thus, the accurate measurement of tHb in emergency, surgery and intensive care settings is vital for understanding the current condition of patients with hemorrhage and is the most important indicator for determining the need for transfusion of RBC products. We previously experienced a case in which hemolysis was observed after infusion or transfusion during veno-arterial extracorporeal membrane oxygenation (VA ECMO), and tHb value differed between conductivity [1] and absorbance [2] measurement methods. Thus, the tHb value did not match the patient’s condition and treatment status.

The conductivity method measures the potential difference between two electrodes in a blood specimen. This potential difference is converted into a hematocrit value according to the characteristic inverse relationship between conductance and the number and size of RBCs in the specimen, and tHb is subsequently calculated. So, the conductivity results regard as the hematocrit. However, this method may be influenced by changes in electrolyte concentration in the absence of tHb changes; therefore, there is doubt concerning the reliability of this method in patients with electrolyte imbalances due to the patient’s condition and the treatment. We should recognize that discrepancy will occur between hematocrit value and tHb in the conductivity method. Alternatively, the absorbance method is not influenced by electrolytic concentration in the absence of tHb changes. However, in the absorbance measurement method, tHb is measured via light absorbance after RBCs are completely hemolysed by ultrasonic waves, so it is impossible to distinguish between hemoglobin from hemolytic RBCs and that released from functional RBCs.

Thus, both measurement methods have advantages and limitations in the clinical setting. Therefore, to examine how conductance- and absorbance-based methods are influenced by electrolyte balance changes and hemolysis, we compared tHb values of blood samples collected from healthy adult volunteers with controlled changes conferred by adding a 10% solution of sodium chloride (NaCl) solution, distilled water (DW) or hemolytic RBCs.

## Methods

### Sample, setting and interventions

**Fig. 1:**
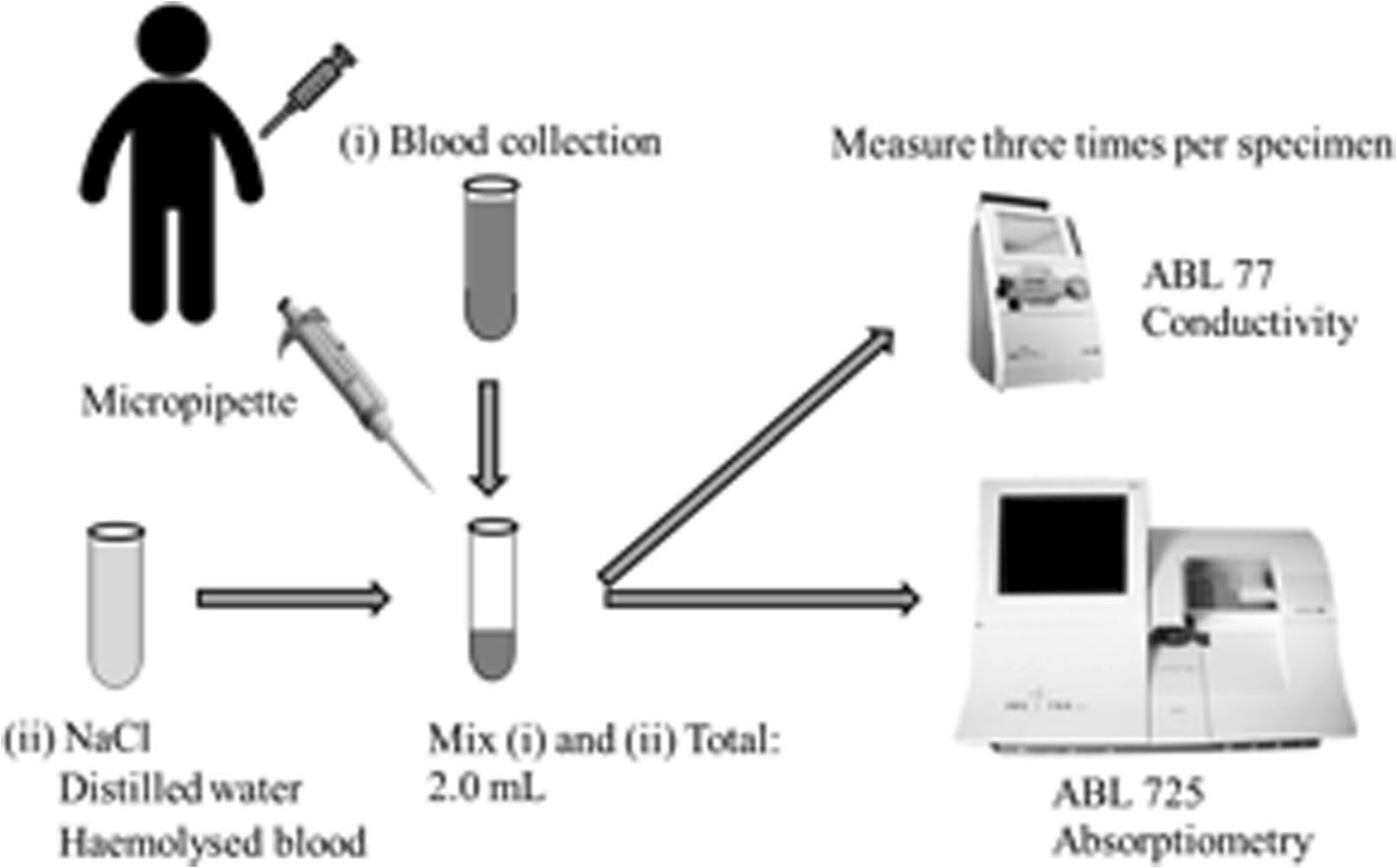
Measured using conductance and absorbance measurements. NaCl solution, DW and hemolysed blood were added to the collected blood

Blood samples were collected from five healthy adult male volunteers (mean age: 33.6 ± 7.40 years, mean height: 172.6 ± 5.26 cm, mean weight: 72.1 ± 3.0 kg), They met the standard blood test of WHO classification (Red blood cell count: 3.87–5.2 × 10^12^/L, White blood cell count: 3.3–8.6 × 10^9^/L, Platelets: 158–348 × 10^9^/L, Hb: 13.7–16.8 g/dl, Hematocrit: 37.4–48.6%, Mean corpuscular volume: 87.2–104.2 fl, Mean hemoglobin content: 29.2–35.3 pg, Mean hemoglobin concentration: 32.0–35.0%) without complications, anemia and hemodyscrasia who provided informed consent to participate in this study. Approximately 18 mL blood per person was collected from the cubitus vein in a vacuum blood vessel using a 21-gauge needle. NaCl solution, DW and hemolysed blood were added to the collected blood from volunteers to create three models, namely, high conductivity, low conductivity and hemolysis, respectively (Fig. 1). Blood tHb value was measured using an ABL 77® (conductivity 77) and ABL 725® (absorbance 725) system for conductivity- and absorbance-based measurements, respectively (both instruments from Radiometer K.K., Tokyo, Japan). For the conductivity change model, 10% NaCl (Otsuka saline 10% solution®; Otsuka Pharmaceutical Co., Ltd., Tokyo, Japan) was added to increase the serum Na^+^ level by 30 and 60 mEq/L (Table. 1), whereas DW (PL®; Fuso Pharmaceutical Industries, Ltd., Tokyo) was added to reduce the serum Na^+^ level by 10 and 20% (Table. 2). DW was added slowly while mixing the sample to prevent hemolysis. Addition of DW did not lead to hemolysis and change mean RBC volume. All samples were stirred to ensure mixing. For the hemolysis model, the collected blood was completely hemolysed by freeze–thawing, and a proportion of this was added to untreated sample. Complete hemolysis was visually confirmed via light microscopy (Table. 3). For the absorbance change model, the clinical spectrophotometer has a measurement range of 478 to 672 nm and uses 128 wavelengths.

**Table 1:** High conductivity model

10%NaCl	Blood volume (ml)	10%NaCl (ml)	Total (ml)
30 mEq/L added	1.975	0.025	2.00
60 mEq/L added	1.950	0.050	2.00

High conductivity model. 10% NaCl was added to increase the serum Na + level by 30 and 60 mEq/L

**Table 2:** Low conductivity model

Distilled water	Blood volume (ml)	Distilled water (ml)	Total (ml)
10% added	1.80	0.20	2.00
20% added	1.60	0.40	2.00

Low conductivity model. DW was added to reduce the serum Na + level by 10 and 20%

**Table 3:** Hemolysed blood model

Hemolysed blood rate(%)	Blood volume (ml)	Hemolysed blood volume (ml)	Total (ml)
10	1.80	0.20	2.00
50	1.00	1.00	2.00
90	0.20	1.80	2.00
100	0.00	2.00	2.00

tHb and Na^+^ levels were measured thrice in each specimen, and each specimen was measured using both methods and tHb corrected for dilution ratio.

### Statistical analysis

All measurements are expressed as mean ± standard deviation, and Student’s *t*-test was used to compare values between the two measurement method groups and between each measurement method group and untreated controls. The level of significance (*P*) was set at 0.05 (two-tailed).

## Results

**Fig. 2:**
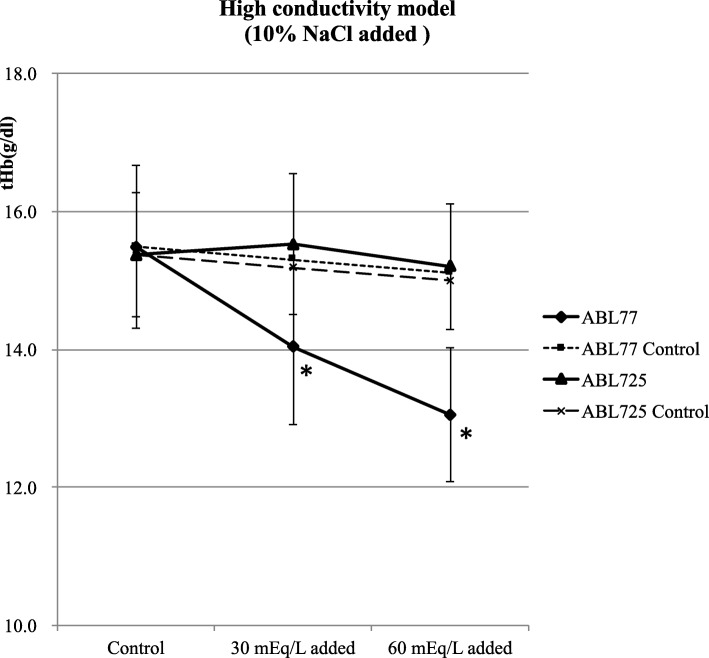
Effect of elevated serum electrolyte concentration (30 and 60 mEq/l Na^+^) on tHb. Mean tHb values significantly differed between control and 10% Nacl added on the conductivity 77. But absorbance 725 showed no difference in tHb values

**Fig. 3:**
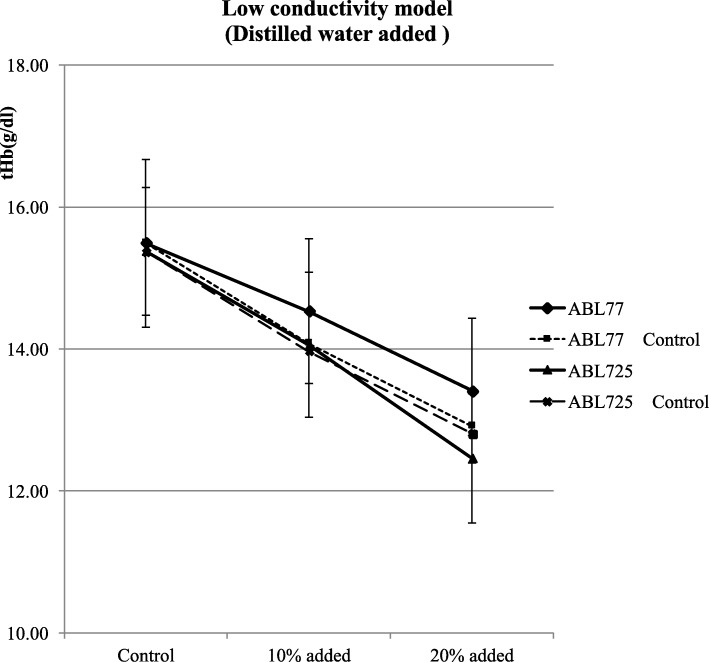
Effect of reduced the serum Na^+^ level by 10 and 20% on tHb. The conductivity 77 and absorbance 725 showed no difference in tHb values

**Fig. 4:**
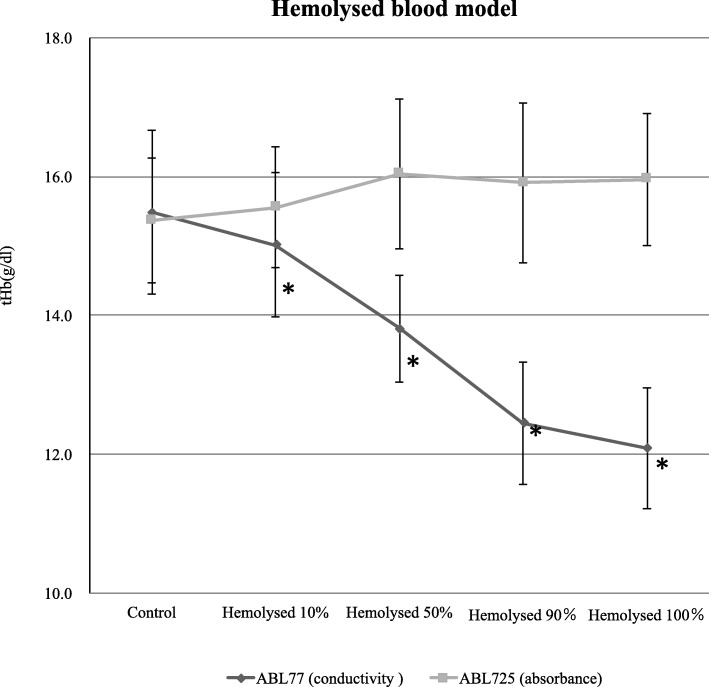
Effect of the hemolysis model (10, 50, 90 and 100% haemolysed) on tHb. Mean tHb values significantly differed between the absorbance 725 and the conductivity 77

**Fig. 5:**
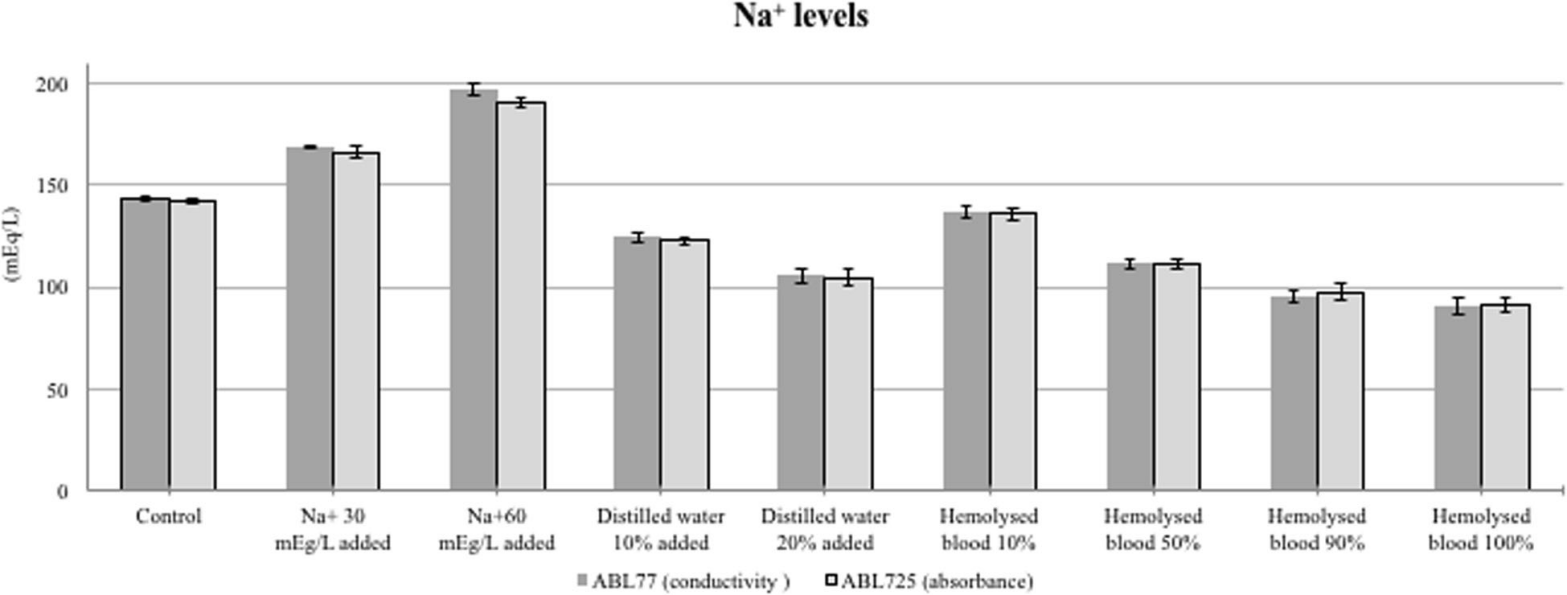
Serum Na^+^ levels among corresponding samples according to conductivity 77 and absorbance 725. The levels were approximately equivalent measurements in all states

Mean tHb values of control (untreated) blood samples did not differ between conductivity 77 and absorbance 725 (15.49 ± 1.18 vs. 15.37 ± 0.09 g/dl, *P* > 0.05). Mean tHb values significantly differed between conductivity and absorbance measurement methods for both the + 30 mEq/ml Na^+^ (14.04 ± 1.13 vs. 15.53 ± 1.02 g/dl, *P* < 0.05) and + 60 mEq/l (13.05 ± 0.97 vs. 15.20 ± 0.91 g/dl, *P* < 0.05) samples. Compared with untreated control samples, the conductivity 77 yielded lower tHb values, whereas the absorbance 725 showed no difference in tHb values (Fig. 2). Thus, conductivity-based but not absorbance-based tHb measurements were sensitive to increased sample electrolyte concentration. Conductivity 77 and absorbance 725 values also significantly differed for the 10% (14.53 ± 1.02 vs. 14.06 ± 0.94 g/dl, *P* < 0.05) and 20% (13.41 ± 1.02 vs. 12.46 ± 0.89 g/dl, *P* < 0.05) dilution samples. Compared with untreated control samples, conductivity 77 yielded elevated tHb values and the magnitude of elevation increased with dilution (20% > 10%), whereas absorbance 725 showed no difference in tHb values (Fig. 3). Conductivity 77 and absorbance 725 values also differed for all hemolysis samples (10%: 15.02 ± 1.04 vs. 15.56 ± 0.87 g/dl, 50%: 13.81 ± 0.77 vs. 16.04 ± 1.08 g/dl, 90%: 12.45 ± 0.88 vs. 15.91 ± 1.15 and 100%: 12.09 ± 0.87 vs. 15.96 ± 0.95 g/dl; all *P* < 0.05). Compared with untreated controls samples, tHb values were progressively reduced by the addition of hemolytic RBCs as measured using conductivity 77, and the values also differed as measured using absorbance 725 (Fig. 4). Serum Na^+^ levels were approximately equivalent among corresponding samples according to conductivity 77 and absorbance 725 measurements (Fig. 5).

## Discussion

Conductivity 77 is a portable blood gas measurement device that operates on the basis of the conductivity principle. The measurement duration is short, and manipulation and maintenance are easy, making conductivity 77 optimal for use in emergencies. The same holds true for the absorbance 725 device. There were no differences between conductivity 77 and absorbance 725 in pH, pCO2, pO2, Na^+^, K^+^, Ca^2+^, and hematocrit, suggesting sufficient reliability. The conductivity method measures the potential difference between two electrodes in a blood specimen. This potential difference is converted into a hematocrit value according to the characteristic inverse relationship between conductance and the number and size of RBCs in the specimen, and tHb is subsequently calculated. So, the conductivity results regard as the hematocrit. However, this method may be influenced by changes in electrolyte concentration in the absence of tHb changes; therefore, there is doubt concerning the reliability of this method in patients with electrolyte imbalances due to the patient’s condition and the treatment. We should recognize that discrepancy will occur between hematocrit value and tHb in the conductivity method. Conductivity 77 is also useful compared with absorbance measurement and centrifugal separation methods. However, a dissociation between conductivity- and absorbance-based hematocrit measurements has been reported [3–5]. Using the conductivity-based method, the measured hematocrit value is lower than the absorbance-based value because of the influence of dilution by infusion, cardiopulmonary bypass and electrolyte concentration [6]. Alternatively, we found that absorbance-based measurements did not differ between control samples (untreated) and samples with experimentally altered electrolyte levels and pre-existing hemolysis (after correcting for dilution).

The conductivity measurement method uses two electrodes that are in direct contact with the specimen blood. A current is passed between the electrodes, and the potential difference is measured. The potential difference varies linearly with conductance, which depends on cell density and electrolyte concentration. However, four factors affect conductance-based measurements aside from cell density/size: however, conductance-based measurements are affected by four factors: electrolyte concentration, protein concentration, osmotic pressure and hemolysis [7, 8].

Blood is a viscous fluid that contains plasma, cells and proteins. Electrolytes account for most of the conductivity of plasma, whereas 99% of the cells are nonconductive RBCs. The measurable Na^+^ level of the conductivity 77 instrument ranges from 80 to 200 mmol/l, and the tHb. value is calculated using Na^+^ correction. In this study, the tHb value was reduced by elevated Na^+^ level (high conductance) and vice versa, suggesting that additional Na^+^ causes errors in tHb measurement because tHb cannot be corrected even within the Na^+^ measurement range of the conductivity 77 instrument.

### Strengths and limitations

Proteins account for 1–7% of the plasma but are nonconductive. Conductivity 77 measures tHb by assuming that the protein concentration is constant. Therefore, in case of excess replacement fluid or low protein levels, the conductivity of blood is increased, which causes an error in tHb measurement [9]. Total osmotic pressure is conferred by crystalloid (mainly from Na^+^) and colloid (mainly from albumin) osmotic pressures. In high osmotic pressure, RBCs volume is reduced. Conversely, low osmotic pressure results in RBCs swelling. In accordance with the principle of the conductivity measurement method, an error may occur because tHb measurement is inversely proportional to RBCs volume. Hemolysis occurs when RBCs (nonconductors) rupture. If protein concentration changes but nothing else does, tHb would change falsely. As the proportion of hemolytic RBCs increases, that of plasma increases. Therefore, our results also suggest that the electrical conductivity of blood increases and measured tHb value decreases with hemolysis.

The absorbance 725 is a desktop-type blood gas measurement device that is based on sample light absorbance. In this case, tHb value is measured according to hemoglobin concentration and optical path length (which are in proportion). Prior to measurement, ultrasonic vibration is applied to completely hemolytic RBCs in the specimen. Therefore, Hb from hemolytic RBCs is added to that from functional RBCs. In our study, electrolytes and hemolysis did not affect absorbance-based tHb measurements. However, fetal hemoglobin (HbF) has been reported to have molecular structure different that is from that of adult hemoglobin, and errors occur in HbF measurement when the total bilirubin concentration is high [10, 11]. By the influence of bilirubin, measurement error occurs on the specimen light absorbance in the wavelength band 478-500 nm. Due to the characteristic difference in the molecular structure between adult Hb (HbA) and HbF, the light absorbance of HbA and HbF are different in the wavelength band of 450 to 500 nm. And, HbF is measured from the difference of absorbance [11, 12]. In severe jaundice the HbA and HbF value measured with the absorbance method are not identical with the real blood concentration of the hemoglobin. HbA and HbF tend to be higher than normal values. In absorbance 725, a bilirubin of 20 mg/ dL can produce an artifact in HbF of 0.5 g/dL [13]. Both instruments also measure Na^+^, but the measurement principle is identical, so there were no differences in the measured values between these instruments.

Based on these findings, when excessive replacement fluid or electrolyte abnormality is suspected, an absorbance-based tHb measurement method should be used. However, absorbance-based measurements should be corrected for Hb from hemolytic RBCs to obtain a better estimate of the blood oxygen carrying capacity.

## Conclusions

Blood gas analysis devices use different principles for tHb measurement. Therefore, the characteristics of the measuring device must be chosen according to potential errors introduced by the pathological state or treatment. For patients suspected of having excessive replacement fluid or an electrolyte abnormality, blood conductivity changes are possible. In such patients, absorbance measurement should be performed while taking hemolysis and bilirubin levels into consideration.

## Data Availability

The datasets used and/or analysed during the current study are available from the corresponding author on reasonable request.

## Abbreviations

tHb: The total hemoglobin
RBCs: Value in red blood cells
VA ECMO: Veno-arterial extracorporeal membrane oxygenation
NaCl: Normal sodium chloride
DW: Distilled water
